# A labelled discrete choice experiment adds realism to the choices presented: preferences for surveillance tests for Barrett esophagus

**DOI:** 10.1186/1471-2288-9-31

**Published:** 2009-05-19

**Authors:** Michelle E Kruijshaar, Marie-Louise Essink-Bot, Bas Donkers, Caspar WN Looman, Peter D Siersema, Ewout W Steyerberg

**Affiliations:** 1 Department of Public Health, Erasmus MC, University Medical Center Rotterdam, Rotterdam, The Netherlands; 2 Department of Business Economics, Erasmus School of Economics, Rotterdam, The Netherlands; 3 Department of Gastroenterology and Hepatology, Erasmus MC, CE Rotterdam, The Netherlands; 4 Respiratory Diseases Department, Centre for Infections, Health Protection Agency, London, NW9 5EQ, UK; 5 Dept. of Social Medicine, Academic Medical Centre, University of Amsterdam, Amsterdam, The Netherlands; 6 Division Internal Medicine and Dermatology, University Medical Center Utrecht, Utrecht, The Netherlands

## Abstract

**Background:**

Discrete choice experiments (DCEs) allow systematic assessment of preferences by asking respondents to choose between scenarios. We conducted a labelled discrete choice experiment with realistic choices to investigate patients' trade-offs between the expected health gains and the burden of testing in surveillance of Barrett esophagus (BE).

**Methods:**

Fifteen choice scenarios were selected based on 2 attributes: 1) type of test (endoscopy and two less burdensome fictitious tests), 2) frequency of surveillance. Each test-frequency combination was associated with its own realistic decrease in risk of dying from esophageal adenocarcinoma. A conditional logit model was fitted.

**Results:**

Of 297 eligible patients (155 BE and 142 with non-specific upper GI symptoms), 247 completed the questionnaire (84%). Patients preferred surveillance to no surveillance. Current surveillance schemes of once every 1–2 years were amongst the most preferred alternatives. Higher health gains were preferred over those with lower health gains, except when test frequencies exceeded once a year. For similar health gains, patients preferred video-capsule over saliva swab and least preferred endoscopy.

**Conclusion:**

This first example of a labelled DCE using realistic scenarios in a healthcare context shows that such experiments are feasible. A comparison of labelled and unlabelled designs taking into account setting and research question is recommended.

## Background

Discrete choice experiments (DCEs) have been proposed as a means to improve systematic assessment of patients' preferences regarding screening and surveillance programs [1]. It is suggested that traditional decision modelling and cost-utility analyses are insufficiently capable of including process effects, such as patient burden, and non-health outcomes, such as information, of diagnostic or therapeutic interventions [2] and that they fail to identify the most optimal program set-up from series of efficient programs [1]. In the end, patient or population preferences determine the acceptance of a program, and hence the realization of the expected population health gains. Current important areas of application include priority setting in health care (e.g., [3]), and patient preferences for characteristics of health care delivery (e.g, [4]) or for treatment alternatives (e.g., [5]).

In a DCE, respondents are asked to choose between different options, each of which is described by a series of attributes at different levels [6-8]. The choices can be presented as labelled ('endoscopy') or unlabelled ('test A'). The majority of published DCEs in health care have used unlabelled designs. Labelled designs offer less abstract choices to respondents and this may add to the validity of the results, although direct comparisons of labelled and unlabelled designs are lacking so far. In both labelled and unlabelled DCEs, the relative importance of the attributes, and the trade-offs made between them, can be assessed using statistical modelling.

The design of the experiment can be orthogonal, without correlations between the attributes, but this may not always be the most statistically efficient design. Non-orthogonal efficient designs can be used to generate designs with dependence between attributes and when the choice probabilities are dependent on the attribute levels [9]. Especially in labelled designs, it may not be possible to prevent such interdependence because, realistically, the levels of one attribute depend on the level of another attribute. For example, choices would be unrealistic if one were to offer cancer screening at different intervals with a lower detection rate for the frequent interval compared to the infrequent one. Such unrealistic choices will reduce respondents' interest and involvement with the questionnaire, and might even lead to invalid choices.

Barrett esophagus (BE) is a condition in which the normal squamous epithelium of the distal esophagus is replaced by columnar epithelium of the intestinal type. Patients with BE are at an increased risk of developing esophageal adenocarcinoma (EAC). Therefore, they are recommended to undergo regular endoscopic surveillance to detect EAC at an earlier stage with more potential for a curative treatment [10]. However, upper GI endoscopy is an invasive procedure that is burdensome to patients and is associated with anxiety and discomfort [11]. Furthermore, the number of patients experiencing this burden is much larger than the number experiencing potential health benefits from it, as the number of patients developing EAC in BE is low (an estimated 0.5% per year) [12-16]. Undisputed evidence that surveillance prolongs survival is not available, although many studies indirectly imply that it is beneficial [17-20].

These data underline the importance of including patients' preferences in recommendations of regular endoscopic surveillance, in addition to evidence on expected health benefit. It is currently unknown, however, how willing patients are to adhere to regular endoscopic surveillance protocols given a specified expected health benefit, if and how they make trade-offs between the burden of testing and expected health benefits, and how their preferences would change if a less invasive test were available.

The present study used a labelled and efficient DCE to investigate patients' and potential future patients' preferences for regular endoscopic surveillance of BE. We assessed the trade-offs patients are willing to make between the burden of testing and the expected health gain when choosing a surveillance scheme, with surveillance schemes differing in test type, test frequency and the resulting expected health benefits.

## Methods

### Respondents

We recruited patients with upper GI endoscopy experience, i.e., patients under regular surveillance for BE and patients with non-specific upper GI symptoms (NS). We approached consecutive patients who had participated in a previous study assessing the burden of upper GI endoscopy who had given permission to be contacted again, within one year after having undergone an upper GI endoscopy [11,21]. The Medical Ethics Committee of Erasmus MC approved the study (MEC 03.1064). In total, 297 patients were eligible; 155 BE patients and 142 NS patients. Inclusion criteria were:

- BE patients: presence of BE of 2 cm or more in length confirmed by histological presence of intestinal metaplasia, absence of high-grade dysplasia and carcinoma, ability to read Dutch and informed consent. These patients were participants to an ongoing trial assessing the value of flow-cytometry in individualizing the frequency of surveillance (CYBAR [22,23]).

- NS patients: referral for endoscopy for non-specific upper GI symptoms, absence of "alarming symptoms", absence of a prior diagnosis of BE, ability to read Dutch and informed consent.

### Questionnaire

#### • Selection of attributes and levels

We selected three potential determinants of patients' preferences for surveillance: the invasiveness of the surveillance test itself, the frequency of testing, and the associated expected health gain. Their relevance as determinants of preferences for endoscopic surveillance was explored and confirmed in individual interviews with 30 BE patients.

We operationalized invasiveness as the 'type of surveillance test', with three 'levels': endoscopy and two fictitious less invasive tests: video capsule endoscopy and a saliva-swab. Although the latter tests are not available for endoscopic surveillance, they are existing tests for other indications and therefore easy to describe and imagine.

Test frequency was presented as the number of times patients would come for endoscopy within the next 10 years, with five levels (in 10 years time: 2, 3, 5, 10, and 40 times, respectively).

The health gain associated with each test-frequency combination was described as the risk of dying from EAC in the next 10 years. We estimated this risk to be 4% in absence of surveillance (loosely based on a one-year risk of detection of EAC of 0.5% [14] and a 5-year survival rate of EAC of 10–20% [24]).

Results from the pilot study showed that patients had difficulties understanding unrealistic scenarios (e.g., increasing test frequency with a more invasive test yielding a lower expected health gain). Therefore, in our experiment a given test that is performed at a given frequency uniquely determined the associated health gain. Table 1 shows the health gains for each combination of test and frequency in the DCE as based on expert opinion. Higher test frequencies result in larger risk reductions, but at a decreasing rate. At the same time, the effectiveness of endoscopy is highest and that of the saliva test lowest, at a given frequency.

**Table 1:** Health gains (remaining 10-year risk of dying from EAC) for each combination of test and frequency in the discrete choice experiment (based on expert opinion and a baseline risk of 4% in the absence of surveillance [14,24,14].

10-year risk of dying (in %) at test frequency					
Frequency (per 10 year):	2	3	5	10	40

Test:					
Saliva swab	3.5	2.5	2.0	1.5	1.2
Video capsule	2.5	2.0	1.5	1.2	1.0
Endoscopy	2.0	1.5	1.2	1.0	0.9

#### • Experimental design

**Figure 1:**
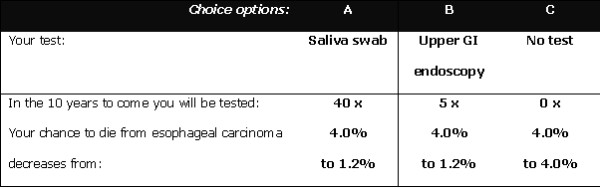
**Example of a choice scenario as presented in the questionnaire**.

We used a labelled design, because the specific aspects of endoscopy that determine its burdensomeness could not be realistically presented to patients using the unlabelled 'test A' -variant. Patients were presented with 15 choices between two scenarios, and additionally had the option to choose not to undergo testing ('opt out'). Figure 1 shows an example of a choice as presented to the respondents. Previous studies have demonstrated that respondents are able to manage up to 16 choices [25,26].

A full-factorial, orthogonal design only provides optimal efficiency in case the attributes do not affect the choice probabilities [9] and does not allow for dependence between the attributes. When non-zero effect sizes are expected, more efficient (so called D-optimal or efficient) designs can be obtained [27]. As we expected non-zero effects of health gains and test frequency and there are clear relationships between the attributes, we estimated a simple linear model based on a small number of choice sets. These estimates served as input for the SAS %ChoicEff macro [28]. This resulted in a near full-factorial efficient design (surveillance by 2 times saliva swab in 10 years was used only once; 5 times saliva swab in 10 years appeared three times) with a random order of appearance.

### Presentation of questionnaire and elicitation mode

Each BE and NS patient received the same 15 choices. The questionnaire started with an introduction explaining the task and the attributes. NS patients were additionally informed about the increased risk of EAC in BE patients, and were asked to fill in the questionnaire as if they themselves had BE.

Three introductory questions were included to help patients to get used to the attributes. Two final items to check the validity of the DCE-results asked patients to express directly which type of test they preferred overall and how often they were reasonably willing to undergo endoscopy at the most. BE patients were furthermore asked to indicate their current surveillance frequency.

Pilot testing of the questionnaire in 3 BE and 4 NS patients showed that the questionnaire was feasible and that patients understood the task. We sent a paper version of the questionnaire to all 297 patients. Follow-up included a telephone call to answer questions, if necessary, and to go through the first three choices together with the respondent in order to assess his (or her) understanding of the task and to find out whether respondents were prepared to make trade-offs.

### Analyses

The Random Utility Model provides the theoretical basis for the analysis of choice data [29]. It assumes that individuals choose the option that yields them the highest utility. As it is unclear how patients trade off health gains against test burden, represented by test type and frequency, we did not impose functional restrictions on this. Instead, each of the possible scenarios was represented with a dummy variable with the no-test option as the reference level. The utility of scenario *j*, *j *= 1,..,15, for respondent *i *is then given by *U*_*ji *_= *U*_*j *_+ *ε*_*ji*_, with *U*_*j *_representing the mean utility for scenario *j *and *ε*_*ji *_the respondent specific deviation from the population mean. Depending on the distributional assumptions on *ε*_*ji*_, the choices of respondents can be modelled using logit or probit specifications. As each question included a choice between three options ('scenarios' – two surveillance alternatives, and a no surveillance option) but only one could be chosen, we used a conditional logit model. The model was used to estimate the utilities, *U*_*j*_, for the 15 test-frequency combinations (as respondents chose one of three alternatives in each choices set it is possible to estimate up to 30 parameters from the answers to 15 choice questions). These utilities have a relative interpretation (i.e., a scenario with a higher utility is preferred over a scenario with a lower utility). The model was estimated using proc PHREG in SAS version 9.1 [30]. Differences between groups (BE or NS) were tested by comparison of the likelihood of the overall model with the sum of the likelihoods of the separate models for BE and NS (likelihood ratio test). Effects of age and sex were studied in the same way.

## Results

### Respondent characteristics and response

A total of 247 patients (133 BE and 114 NS) returned completed questionnaires (response rate 84%). The mean age of the respondents was 59 years, and the majority was either employed (39.9%) or retired (43.2%) (Table 2). BE patients were more often male than female. Most BE patients underwent surveillance endoscopy once every two years (86.9%). We were able to contact 71% of the respondents (175/247) in the telephone follow-up. Some of the patients wanted additional information or needed help with the task, but most had already filled in the questionnaire (n = 109) or preferred to do this by themselves (n = 30). All interviewed patients seemed to understand the task and made trade-offs.

**Table 2:** Characteristics of participants

Variable	All patients	BE patients	NS patients	N
Group	247 (100%)	133 (53.8%)	114 (46.2%)	247

Hospital of origin:				247
- A	26 (10.5%)	26 (19.5%)	0	
- B	39 (15.8%)	0	39 (34.2%)	
- C	47 (19.0%)	47 (35.3%)	0	
- D	135 (54.7%)	60 (45.1%)	75 (65.8%)	

Mean age (sd)	59 (13)	62 (11)	56 (14)	247

Sex: male	141 (57.1%)	84 (63.2%)	57 (50%)	247

Civil status:				246
- married/living together	197 (80.1%)	108 (81.8%)	89 (78.1%)	
- never married	18 (7.3%)	8 (6.1%)	10 (8.8%)	
- divorced	15 (6.1%)	6 (4.5%)	9 (7.9%)	
- widowed	16. (6.5%)	10 (7.6%)	6 (5.3%)	

Employment				243
- paid work	97 (39.9%)	44 (33.3%)	53 (47.7%)	
- no/unpaid	41 (16.9%)	22 (16.7%)	19 (17.1%)	
- retired	105 (43.2%)	66 (50%)	39 (35.1%)	

Education				242
- elementary	37 (15.3%)	21 (16.3%)	16 (14.2%)	
- secondary	149 (61.6%)	77 (59.7%)	72 (63.7%)	
- tertiary	56 (23.1%)	31 (24.0%)	25 (22.1%)	

Endoscopic surveillance: once every two years	N.a.	91 (86.9%)	N.a.	132

### Preferences for test-frequency combinations

Table 3 shows the estimated utilities of the 15 scenarios that were evaluated. All tests-frequency combinations have a positive utility, indicating that they are preferred over 'no test' and these effects were all significant (p < 0.01). The most preferred test was the video test at a frequency of once a year, which achieved the highest estimated utility of 6.03. This utility is significantly larger than all other utilities, except compared to endoscopy every one or two years. The combinations of endoscopy every one or two years had the next highest utilities and were significantly preferred over all other tests except video capsule every one or two years. The p-values for pair-wise comparisons of the utilities of all test-frequency combinations are given in Table 4. For example, an increase in frequency of endoscopy from once every two years to each year, with an associated increase in expected health gain, did not result in a significant increase in utility (p = 0.85).

**Table 3:** Estimated utility of test-frequency combinations (reference = no surveillance).

	Coefficients (standard error)				
Frequency (per 10 year):	2	3	5	10	40

Test:					
Saliva swab	0.82 (0.32)^∞^	3.07 (0.22)*	3.99 (0.24)*	5.07 (0.29)*	5.32 (0.27)*
Video capsule	3.20 (0.22)*	4.55 (0.23)*	5.57 (0.28)*	6.03 (0.30)*	5.18 (0.29)*
Endoscopy	3.56 (0.24)*	4.89 (0.28)*	5.86 (0.30)*	5.89 (0.30)*	4.53 (0.30)*

* significantly different from no surveillance, p < 0.0001, ∞ p = 0.01

**Table 4:** Significance (p-values) for pair-wise comparisons of the utilities of all test-frequency combinations.

	Saliva swab 2×	Saliva swab 3×	Saliva swab 5×	Saliva swab 10×	Saliva swab 40×	Video capsule 2×	Video capsule 3×	Video capsule 5×	Video capsule 10×	Video capsule 40×	Endoscopy 2×	Endoscopy 3×	Endoscopy 5×	Endoscopy 10×	Endoscopy 40×
Saliva swab 2×	-														
Saliva swab 3×	< .0001	-													
Saliva swab 5×	< .0001	< .0001	-												
Saliva swab 10×	< .0001	< .0001	< .0001	-											
Saliva swab 40×	< .0001	< .0001	< .0001	**0.24**	-										
Video capsule 2×	< .0001	**0.29**	0.00	< .0001	< .0001	-									
Video capsule 3×	< .0001	< .0001	0.00	0.04	0.00	< .0001	-								
Video capsule 5×	< .0001	< .0001	< .0001	< .0001	**0.22**	< .0001	< .0001	-							
Video capsule 10×	< .0001	< .0001	< .0001	< .0001	0.00	< .0001	< .0001	0.01	-						
Video capsule 40×	< .0001	< .0001	< .0001	**0.62**	**0.39**	< .0001	0.01	**0.08**	< .0001	-					
Endoscopy 2×	< .0001	0.01	0.00	< .0001	< .0001	**0.11**	0.01	< .0001	< .0001	< .0001	-				
Endoscopy 3×	< .0001	< .0001	< .0001	**0.40**	0.00	< .0001	< .0001	0.00	< .0001	0.02	< .0001	-			
Endoscopy 5×	< .0001	< .0001	< .0001	< .0001	0.01	< .0001	< .0001	**0.16**	**0.30**	0.00	< .0001	< .0001	-		
Endoscopy 10×	< .0001	< .0001	< .0001	< .0001	0.01	< .0001	< .0001	**0.10**	**0.23**	0.00	< .0001	< .0001	**0.85**	-	
Endoscopy 40×	< .0001	< .0001	0.01	0.01	< .0001	< .0001	< .0001	< .0001	< .0001	< .0001	< .0001	0.03	< .0001	< .0001	-

Bold p-values highlight non-significant differences

**Figure 2:**
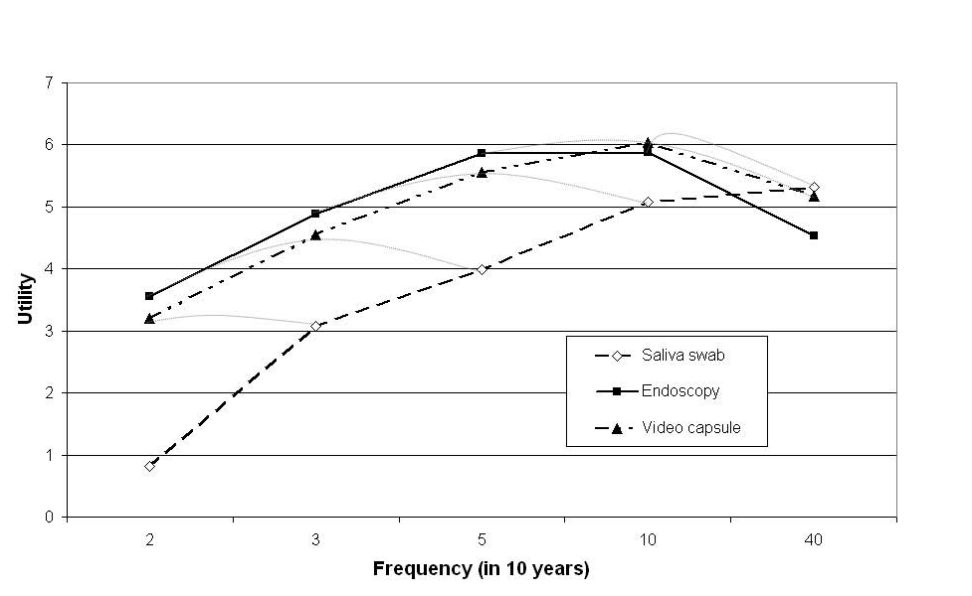
**Utility of endoscopic surveillance by test and frequency (reference = no surveillance, the x-axis)**.

Figure 2 shows a graphical presentation of the results. The horizontal zero utility line (x-axis) refers to not being tested. The picture visualizes that higher test frequencies which are associated with larger health gains are more desirable than lower frequencies, up to a certain threshold. For the video capsule and endoscopy, which are more invasive than a saliva swab, raising test frequency to once a year or more was associated with a decline in utility.

The results also indicate that for the same health gain (which are shown as the bridging lines in Figure 2), patients preferred video capsule over saliva swab, and least preferred endoscopy (p < 0.05). For example, the utility of video capsule 3x is larger than saliva swab 5x, and endoscopy 2x, while all have the same health gain (remaining risk of dying from EAC: 2%, see Table 1). However, this only holds for test frequencies up to once every year. For a decrease in the risk of dying form EAC from 4.0% to 1.2%, which in our experiment could be achieved by saliva swab 40x, video capsule 10x and endoscopy 5x, endoscopy was preferred over saliva swab (p = 0.01, Table 4). At a risk of dying from EAC of 1%, annual endoscopy is also preferred over quarterly tests with the video capsule (p = 0.00).

### Effects of patient characteristics

**Figure 3:**
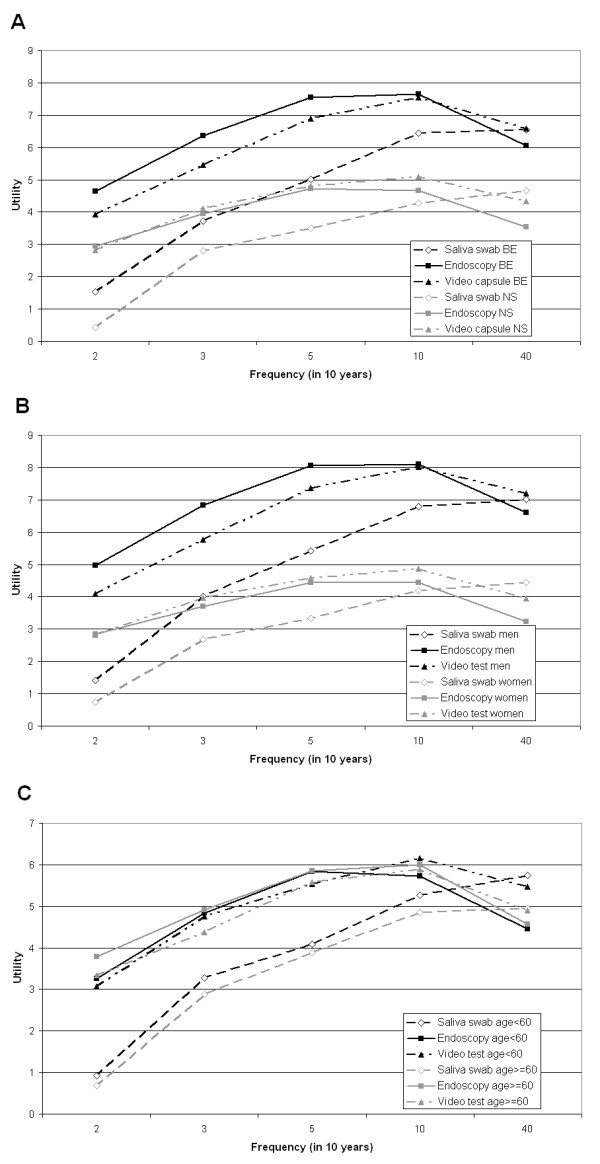
**A. Utility of endoscopic surveillance tests by patient type**. B. Utility of endoscopic surveillance tests by gender. C. Utility of endoscopic surveillance test by age-group. BE: patients with Barrett esophagus. NS: patients with non-specific upper GI symptoms.

The model significantly improved when preferences were estimated separately for BE and NS patients, for men and women and for patients older or younger than 60 years (likelihood ratio tests, all p-values < 0.001). Figure 3A shows that BE patients assigned a larger utility to all test-frequency combinations than NS patients. This difference was significant for all scenarios (p < 0.05) except for a saliva swab once every 5 or 3 years.

Men also gave all test-frequency combinations a higher value than women (Figure 3B). The gender difference was significant for all scenarios (p < 0.05), except for saliva swab once every 5 years. Younger patients seemed to give a higher utility to the saliva swab at any frequency compared to patients older than 60, but this difference was not statistically significant (p > 0.3), and not observed for the other tests.

Despite the higher utility assigned by BE patients and by men, the pattern of utilities was similar; with higher frequencies being preferred over lower ones (up to a certain point), and saliva swab being the least preferred. The patterns differed in the fact that BE patients and men preferred endoscopy over the video capsule for testing up to once a year and NS patients and women only for testing up to every 3–5 years.

### Direct preferences (validity tests)

The responses to the direct items showed that the video capsule test was preferred by more patients (n = 103, 43%) than the saliva test (n = 65) and endoscopy (n = 60). Twelve patients did not express a clear preference. The maximum acceptable frequency of endoscopy from the direct expressions was once a year (n = 122, 50%); or once every two years (n = 84, 35%) for the majority of patients. Only a few of the BE patients were willing to undergo endoscopy more often (n = 15) or less often (n = 21).

## Discussion

### Main findings

This example of an analysis of patients' preferences for surveillance of BE shows that using a labelled DCE with dependency between attributes can add realism and is feasible. Patients were prepared to make trade-offs between the burden of surveillance and the expected health gain, and preferences were supported by comparison with direct preference questions. The findings indicate that the current endoscopic surveillance schemes of once every one or two years were amongst the most preferred alternatives amongst patients, suggesting these are well accepted.

### Strengths and weaknesses

This is, to the best of our knowledge, the first labelled DCE in a health care setting that uses realistic scenarios associating each test-frequency combination with a specific expected health gain. The great advantage of this design is that it adds to the realism of the choices as presented to the patients, and hence to the validity of the results. Using a labelled design was deemed necessary as we considered it impossible to convey the essential elements of the burden of various tests without naming the test. Endoscopy requires patients to swallow an instrument, which can be associated with unpleasant sensations, such as belching, nausea and vomiting. Labelling makes it possible to take all essential elements into account, while an unlabeled experiment requires each of these to be named separately as an attribute (e.g. invasiveness, belching, nausea, vomiting, etc). Moreover, each would have to be estimated separately, requiring a large number of observations, and unrealistic choices would be generated (e.g. a non-invasive test that causes vomiting).

We also considered it essential to fix the health gain associated with a certain test-frequency combination, as results from a pilot study showed that patients had difficulties understanding choices in which health gain was counter-intuitively. For example, respondents tended to choose a scenario with more frequent testing because this would incur a larger health gain, despite the scenario stating a lower health gain. It clearly was not possible to generate valid responses without associating health gain with test-frequency. However, this also has some disadvantages. First, it means that the attributes are correlated and should not be analysed separately. We therefore did not generate preferences for each attribute, but for each test-frequency combination, thereby respecting the co-dependency. We could thus not disentangle the utility associated with the health gain from the attribute-specific utilities associated with the invasiveness of the test and the test frequency. Second, it limits the generalizability of our results, as patients are likely to make different choices when a different health gain would have been associated with the scenarios. The baseline risk of dying of EAC in the absence of surveillance of 4% in 10 years, however, is realistic for most Barrett patients [31]. The results of this study may not hold for BE patients with high-grade dysplasia who are at a higher risk of (dying from) EAC. Finally, using uniquely determined health gains meant that we could not check patients' understanding of the DCE using a so-called 'dominant option' (a choice including one scenario that is logically preferable at the level of all attributes). The results of the direct preference assessment items, nevertheless, validated the results; the video capsule was the test most frequently preferred by patients in the direct statements, and the most preferred endoscopy frequency was once every one or two years.

This is also the first report of a systematic assessment of patients' preferences for surveillance schemes. Subjects had to make explicit choices between different test-frequency combinations, each of them with their corresponding health gain. In the 30 qualitative patient interviews used to select the attributes and their levels, many patients mentioned their tendency to follow their doctor's advice and not being used to make deliberative choices. Although all could well imagine the trade-offs in the DCE, preferences may therefore have been constructed by the DCE for some respondents, instead of the DCE measuring pre-existing preferences [32]. However, this limitation is not specific for our study but is applicable to every formal preference assessment. In fact, we took attempts to make the choice options as realistic as possible. Furthermore, NS patients also preferred endoscopy once every one or two years, without being aware these are the recommended frequencies.

### Preferences for BE surveillance

Patients preferred being tested over not being tested. Moreover, they preferred test-frequency combinations with larger health gains, but for the more burdensome tests, this was only up to a certain frequency of testing. When the health gain was assumed to be similar, patients preferred video-capsule over saliva swab and least preferred endoscopy. Although we cannot provide separate estimates for the utility attributed to a certain health gain or the utility of more frequent testing, it seems sensible to suggest that more frequent testing and more burdensome tests were preferred because of the higher health gain incurred. Patients thus seem to optimise the health gain incurred by more frequent and burdensome endoscopic surveillance, up to a frequency of testing of around once a year. The health gain associated with more frequent testing than once a year does not outweigh the disutility of such frequent testing.

We found some differences between respondent groups. BE patients, who are under regular surveillance, attributed more utility to all tests-frequency combinations than patients with non-specific GI complaints. This means that, given the same test, more BE patients would choose to undergo the test than NS patients. This may relate to BE patients having a better understanding of the feelings and/or risks associated with having BE. The different age-distribution of the respondent groups is unlikely to explain the difference as the analysis by age did not show the same pattern. Men also attributed more utility to being tested then women, which might be attributed to different risk attitudes of men and women.

## Conclusion

This study shows that in the health care setting the application of labelled DCEs adds realism and is feasible. Further realism can be added by creating dependency between some of the attributes. Although this might be required if patients cannot cope with more complex choices, it comes at the cost of analytical power.

This study furthermore indicates the importance of research into the effectiveness of endoscopic surveillance for patients with BE, and into reducing the test burden, for example by optimising the test-frequency based on clinical characteristics.

## Competing interests

The authors declare that they have no competing interests.

## Authors' contributions

MK carried out the questionnaire studies, contributed to the data analysis and study design and draft the paper. ME conceived of the study, and participated in its design, coordination and interpretation, and helped to draft the paper. BD contributed to the study design, analysed the data and drafted technical parts of the text. CL participated in the analysis and interpretation of the paper. PS contributed to the study design and coordination and commented on the text. ES participated in the study design, coordination and interpretation and commented on the text.

## Pre-publication history

The pre-publication history for this paper can be accessed here:

http://www.biomedcentral.com/1471-2288/9/31/prepub
